# Mutations in *P. falciparum* K13 propeller gene from Bangladesh: emerging resistance?

**DOI:** 10.1186/1475-2875-13-S1-P71

**Published:** 2014-09-22

**Authors:** Mohammed Shafiul Alam, Abu Naser Mohon, Abebe Bayih, Asongna Folefoc, Dylan Pillai

**Affiliations:** 1 University of Calgary, Calgary, AB, Canada; 2 ICDDR, B, Dhaka, Bangladesh

## 

Bangladesh is a malaria hypo-endemic country sharing borders with India and Myanmar. ACT remains quite successful in Bangladesh. An increase of heritable artemisinin resistant malaria parasites on the Thai-Cambodia border and subsequently the Thai-Myanmar border is worrisome. K13 propeller gene (PF3D7_1343700 (PF13_0238) mutations have been linked to both *in vitro* artemisinin resistance and *in vivo* slow parasite clearance rates and therefore proposed as a marker of artemisinin resistance. We evaluated if mutations seen in Cambodia have emerged in Bangladesh where ACT use is now standard for over a decade. Samples were obtained from *P. falciparum* infected malaria patients from Upazila Health Complexes (UHC) of seven endemic districts of Bangladesh. These districts included Khagrachhari (Matiranga UHC), Rangamati (Rajsthali UHC), Cox’s Bazar (Ramu and Ukhia UHC), Bandarban (Lama UHC), Mymensingh (Haluaghat UHC), Netrokona (Durgapur and Kalmakanda UHC) and Moulvibazar (Srimongol and Kamalganj UHC). Out of 296 microscopically positive *P. falciparum* samples, 271(91.6%) were confirmed by both real-time PCR and nested PCR to have pure infection. Nested PCR of the K13 gene amplified 253 (93.4%) samples. After ClustalW alignment and Jalview analysis, we initially found three non-synonymous mutations (A578S, W470C, and Y604H) in Bangladeshi clinical isolates. The A578S mutation was confirmed and lies adjacent to the C580Y mutation, the major mutation observed in Cambodia, and based on computational modeling should have a significant effect on tertiary structure. We conclude that novel mutations that may confer artemisinin resistance are present in certain districts of Bangladesh.

